# Corneal endothelial cell density and its correlation with birth weight, anthropometric parameters, and ocular biometric parameters in Chinese school children

**DOI:** 10.1186/s12886-022-02561-1

**Published:** 2022-08-06

**Authors:** Zijin Wang, Xiaoxia Zuo, Lei Liu, Xuejuan Chen, Rui Li, Hui Zhu, Dan Huang, Haohai Tong, Xiaoyan Zhao, Wen Yan, Shiya Shen, Yun Wang, Xiaoxiao Li, Andi Zhao, Danni Chen, Ranran Ding, Shiding Li, Hu Liu

**Affiliations:** 1 Department of Ophthalmology, The First Affiliated Hospital With Nanjing Medical University, 300 Guangzhou Road, Nanjing, 210029 China; 2 Department of Child Healthcare, The First Affiliated Hospital With Nanjing Medical University, Nanjing, China; 3 Eye Center, The Second Affiliated Hospital, Zhejiang University School of Medicine, Hangzhou, China; 4 Department of Ophthalmology, the Affiliated Changzhou No.2 People’s Hospital of Nanjing Medical University, Changzhou, China; 5 Department of Ophthalmology, The Fourth Affiliated Hospital of Nantong University, Yancheng, China; 6 Department of Ophthalmology, Children’s Hospital of Soochow University, Suzhou, China; 7 The Fourth School of Clinical Medicine, Nanjing Medical University, Nanjing, China

**Keywords:** Corneal endothelial cell density, Epidemiology, Birth weight, Body mass index, Ocular parameters

## Abstract

**Background:**

To describe the distribution of corneal endothelial cell density (ECD), and to explore its correlation with birth weight (BW), anthropometric parameters, and ocular biometric parameters in Chinese school children.

**Methods:**

In the population-based cross-sectional Nanjing Eye Study, children were measured for anthropometric information, for ECD by the noncontact specular microscope and for ocular biometric parameters by the optic low-coherent reflectometer. Data from right eyes were analyzed to illustrate the distribution of ECD and for determining correlated factors with ECD using univariate and multiple linear regression analysis. Comparisons among three different BW groups were performed using a one-way ANOVA analysis followed by the Bonferroni correction for pairwise comparisons.

**Results:**

Of 1171 children, the mean (± standard deviation) ECD was 2875.34 ± 195.00 cells/mm^2^. In the Multiple Linear Regression analysis, BW, gender and central corneal thickness were significantly associated with ECD. The ECD increased by 36.16 cells/mm^2^ with BW increasing by 1 kg (*P* = 0.001) and increased by 0.44 cells/mm^2^ for every additional 1 mm in central corneal thickness (*P* = 0.01). The ECD of girls was 54.41 cells/mm^2^ higher than boys (*P* < 0.001). Children born with low BW presented significantly lower ECD than those born with normal BW (*P* < 0.05) and high BW (*P* < 0.05). Age and axial length were not significantly associated with ECD (*P* = 0.06 and *P* = 0.21, respectively).

**Conclusions:**

In Chinese school children aged 82 to 94 months, the ECD is positively correlated with BW and central corneal thickness, in which BW is a newly identified associated factor. It is like that gender plays an important role in ECD distribution while girls have relatively greater ECD than boys.

## Background

The corneal endothelium is the innermost layer of the cornea adhering to Descemet’s membrane, which acts at maintaining corneal transparency by regulating fluid and solute transport between the aqueous humor and corneal stroma [1]. It is well recognized that corneal endothelial cells cannot regenerate under normal circumstances and the density decreases with age at different rates, within different periods and within different individuals [2–6]. Ocular factors, such as trauma, intraocular surgery, glaucoma, wearing contact lens and dry eye, may lead to quality and quantity reduction of corneal endothelial cells [7–9]. Other related factors have been reported to be systemic diseases such as diabetes mellitus and unhealthy habits such as smoking [10–13]. However, factors that influence corneal endothelium cell density (ECD) of normal young individuals are still elusive. Previous studies have evaluated the ECD distribution in different populations [14–27], but most of the subjects were adults.

The purpose of this study was to describe the ECD distribution, and to determine the correlation between ECD and birth weight (BW), anthropometric parameters, and ocular biometric parameters within narrow age range in Chinese school children in the Nanjing Eye Study (NES).

## Materials and methods

### Study design and subjects

The NES is an ongoing population-based open cohort study, designed to longitudinally observe the onset and progression of childhood ocular diseases in eastern China [28–31]. The study was approved by the institutional review board in the First Affiliated Hospital with Nanjing Medical University and was conducted in accordance with the tenets of the Declaration of Helsinki. Informed consent was obtained from the parents or guardians of all children in the study. The study population for the present study consisted of 82 to 94-month-old children enrolled in primary schools in the Yuhuatai District of Nanjing City in East China. Data from eye examinations and questionnaire presented in this paper were collected in 2019.

### Eye examination and questionnaire

Anthropometric information was collected, including height (m), weight (kg), and body mass index (BMI) calculated as weight/height^2. All children underwent comprehensive eye examinations, including slit-lamp examination of the anterior segment, evaluation of ECD by the noncontact specular microscope (Topcon SP-1P; Topcon Corporation, Tokyo, Japan) and measurement of ocular biometric parameters by the optic low-coherent reflectometer (IOLMaster-500; Carl Zeiss Meditec AG, Jena, Germany). ECD is the number of cells present in a 1-mm^2^ area. The measurements of ECD were taken automatically over an approximate area of 0.3 mm^2^ covering the central region of the corneal apex [32] using the panorama photography mode. The panorama mode takes three images in the central and the adjacent nasal and temporal areas. Then the images are automatically combined to create a larger area for the observation and analysis of endothelial cells. Three successful measurements were taken and the ECD results were averaged to obtain a mean value. Ocular biometric parameters by IOLMaster include central corneal thickness (CCT), corneal radius (CR), axial length (AL) and white-to-white corneal diameter (WTW). The surface area of cornea (SAC) was calculated approximately by considering cornea to be spherical and using the area formula for the spherical cap [33]:

$$S=2 \times \pi \times R \times h$$

where *S* is the surface area of the spherical cap; *R* is the radius of the sphere and *h* is the height of the spherical cap.

SAC calculation was derived from the aforementioned formula:

$$R = CR$$

$$h = CR - CR \times cos (arcsin (1/2 \times WTW/CR))$$

$$SAC = 2 \times \pi \times CR \times (CR - CR \times cos (arcsin (1/2 \times WTW/CR)))$$

The BW of each child was obtained from the questionnaire distributed to the legal guardians, which partly represents the birth condition of the children. The BW was categorized as low (< 2.5 kg), normal (2.5–4 kg) and high (> 4 kg).

## Statistical analysis

The Statistical Package for the Social Sciences (R V.4.0.5, 2021–03-31, R Foundation for Statistical Computing, http://www.cran.r-project.org/) was conducted for all statistical analyses. Results were presented as mean ± standard deviation (SD) and 95% confidence interval (CI) for continuous measures, as percentage and 95% CI for categoric measurements. The independent sample t-test was used to assess the primary association between ECD and gender. Pearson's correlation and Univariate Linear Regression (ULR) were applied to test for the significance of the associations between ECD and continuous variables: BW, age, BMI, AL, CR, WTW, CCT and SAC. When significant associations (*P* < 0.05) between ECD and the aforementioned variables were found, these variables were put into the Multiple Linear Regression (MLR) model. We checked assumptions of linear regression through the normal probability plot, Leven test and Kolmogorov–Smirnov non-parametric test. Multicollinearity was assessed by calculating the variance inflation factor. Comparisons among three different BW groups were performed using a one-way ANOVA analysis followed by the Bonferroni correction for pairwise comparisons. Statistical significance was interpreted as *P*-value < 0.05.

## Results

### Characteristics of study population

A total of 1565 children were examined for ECD and ocular biometric parameters, among which 319 children’s corneal endothelial image from right eyes didn’t reach the standard after repeated attempts or the ocular biometric parameters involved were not complete from right eyes. Guardians of 75 children did not complete the questionnaire, leaving 1171 children included in this study.

The mean (± SD) age of was 7.38 ± 0.29 years and 636 (54.3%) participants were boys. The mean (± SD) ECD was 2875.34 ± 195.00 cells/mm^2^, ranging from 2066 to 3546 cells/mm^2^. The characteristics for ECD, BW, gender, age, BMI, and ocular parameters are shown in Table 1.

**Table 1:** Distribution of corneal endothelial cell density, general condition, anthropometric parameters, ocular biometric parameters and birth weight

	**Overall**	**95% Confidence Coefficient**	**Minimum**	**Maximum**
^a^**ECD (mean** ± **SD) (cells/mm**^**2**^**)**	2875.34 ± 195.00	2864.16–2886.52	2066.00	3546.00
**Gender: male (%)**	636 (54.30)	51.41–57.19		
**Age (mean** ± **SD) (year)**	7.38 ± 0.29	6.83–7.83	6.50	8.08
^b^**BMI (mean** ± **SD) (kg/m**^**2**^**)**	16.43 ± 2.69	16.28–16.58	9.22	38.41
^c^**CCT (mean** ± **SD) (mm)**	546.57 ± 33.02	544.68–548.46	427.00	645.00
**Axial Length (mean** ± **SD) (mm)**	22.98 ± 0.79	22.93–23.02	19.25	26.53
^d^**WTW (mean** ± **SD) (mm)**	12.14 ± 0.38	12.12–12.17	10.50	13.20
**Corneal Radius (mean** ± **(SD)) (mm)**	7.81 ± 0.25	7.79–7.82	7.02	8.76
**Birth Weight (mean** ± **(SD)) (kg)**	3.31 ± 0.51	3.28–3.34	1.25	4.50

^a^***ECD*** corneal endothelial cell density;

^b^***BMI*** body mass index;

^c^***CCT*** central corneal thickness;

^d^***WTW*** white-to-white corneal diameter

**Fig. 1:**
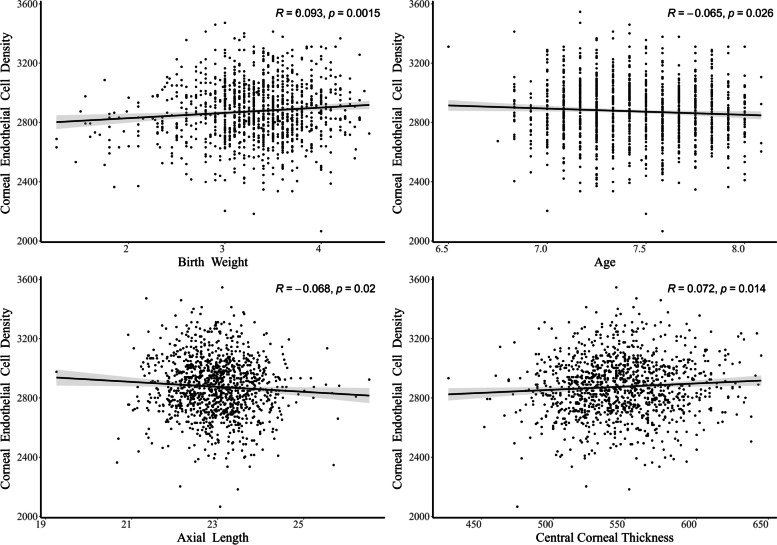
(Upper left) the scatter plot of corneal endothelial cell density versus birth weight. (Upper right) the scatter plot of corneal endothelial cell density versus age. (Lower left) the scatter plot of corneal endothelial cell density versus axial length. (Lower right) the scatter plot of corneal endothelial cell density versus central corneal thickness

**Fig. 2:**
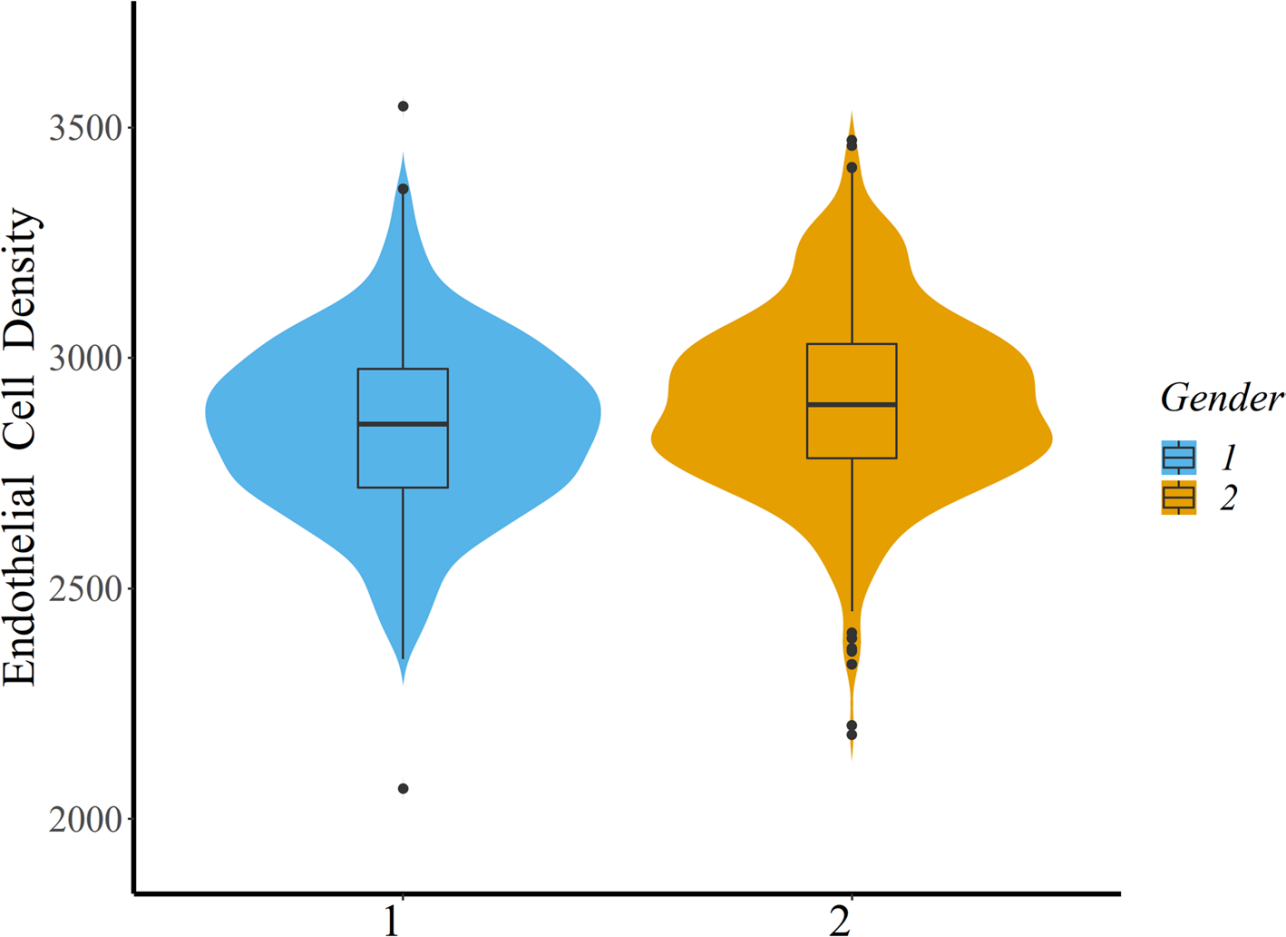
Violin plot combined with box plot analysis comparing the distribution of corneal endothelial cell density (ECD) in the boys and girls. The blue group represents boys (Group 1), whereas the orange group represents girls (Group 2). The mean (± SD) ECD in boys was 2850.31 ± 188.57 cells/mm^2^ and the mean (± SD) ECD in girls was 2905.10 ± 198.48 cells/mm^2^

Pearson’s correlation coefficient demonstrated significant correlation between ECD and BW (*R* = 0.09, *P* = 0.002). There was significant correlation between ECD and age (*R* = -0.07, *P* = 0.03). Girls had relatively greater ECD than boys (*P* < 0.001). Whereas, there was no significant correlation between ECD and BMI (*P* = 0.40). Among all the ocular biometric parameters, ECD had no significant correlation with WTW (*P* = 0.49) and CR (*P* = 0.65), while negative correlation was found between ECD and AL (*R* = -0.07, *P* = 0.02) and positive correlation was found between ECD and CCT (*R* = 0.07, *P* = 0.01). Though we calculated SAC to explore its relationship with ECD, no statistically significant correlation was demonstrated (*P* = 0.47). Figure 1 shows the relationship between ECD and the relatively significantly correlated continuous variables. Figure 2 displays ECD distribution in boys and girls.

**Fig. 3:**
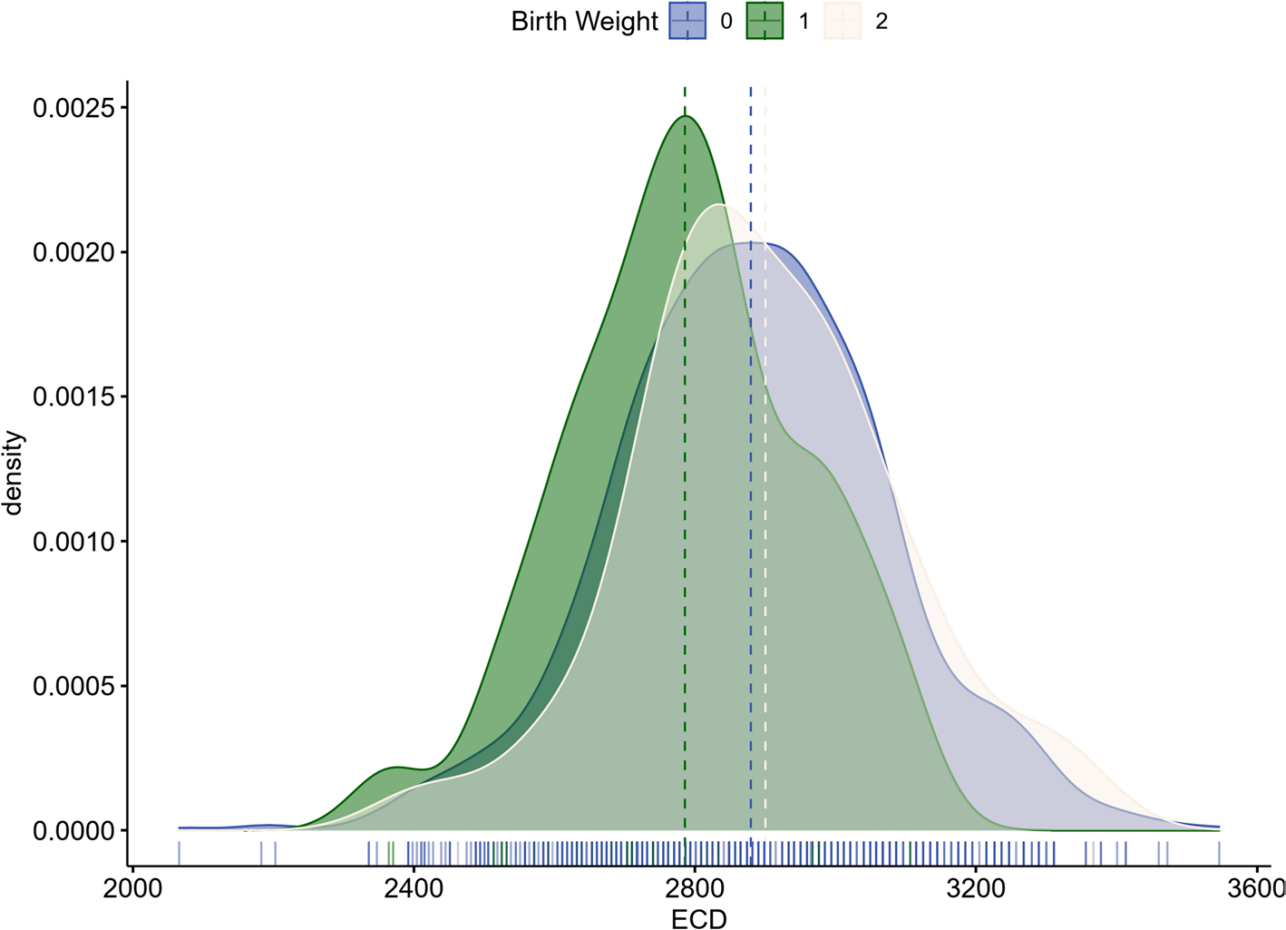
Density plot of corneal endothelial cell density (ECD) in low birth weight group (Group 1), normal birth weight group (Group 0) and high birth weight group (Group 2). The mean ECD was 2785.81 cells/mm^2^ in low birth weight group, 2879.71 cells/mm^2^ in normal birth weight group and 2899.82 cells/mm^2^ in high birth weight group

Table 2 lists the results from the ULR and MLR models. Five variables which were significantly associated with ECD in the ULR model were put into the MLR model. In the MLR analysis, three variables remained significantly associated with ECD: BW, gender and CCT. Age and AL were no longer associated with ECD significantly (*P* = 0.06 and *P* = 0.21, respectively). The ECD increased by 36.16 cells/mm^2^ with BW increasing by 1 kg (*P* = 0.001). The ECD of girls were 54.41 cells/mm^2^ higher than boys (*P* < 0.001). The ECD was positively associated with CCT (*P* = 0.01) while the ECD increased by 0.44 cells/mm^2^ with CCT increasing by 1 mm. To further verify the relationship between BW and ECD, we plotted the distribution of ECD among the groups categorized by BW (Fig. 3). The mean (± SD) ECD was 2785.81 ± 167.42 cells/mm^2^ in low BW group, 2879.71 ± 195.55 cells/mm^2^ in normal BW group and 2899.82 ± 191.83 cells/mm^2^ in high BW group. Comparisons among the three groups were performed using the one-way ANOVA analysis, which showed significant difference (*P* < 0.001). Pairwise comparisons by Bonferroni correction demonstrated that ECD in children born with low BW was significantly lower than that in children born with normal BW (*P* < 0.05) and high BW (*P* < 0.05). However, the normal BW group and high BW group didn’t show significant difference for ECD.

**Table 2:** The univariate and multivariate linear regression models for corneal endothelial cell density

Variables	Univariate Linear Regression			Multivariate Linear Regression		
	β	95% CI	*P*	β	95% CI	*P*
Gender (female vs. male)	54.79	32.58 ~ 77.00	< 0.001*	54.41	31.39 ~ 77.53	< 0.001*
Age	-42.30	-79.60 ~ -5.00	0.03*	-36.1	-73.03 ~ 0.83	0.06
^a^CCT	0.43	0.09 ~ 0.76	0.01*	0.44	0.11 ~ 0.77	0.01*
Axial Length (mm)	-16.72	-30.83 ~ -2.60	0.02*	-9.42	-24.01 ~ 5.18	0.21
Birth Weight (kg)	35.67	13.72 ~ 57.62	0.002*	36.16	14.27 ~ 58.05	0.001*
^b^BMI (kg/m^2^)	-1.77	-5.92 ~ 2.38	0.40			
^c^WTW (mm)	-10.54	-35.21 ~ 19.13	0.49			
Corneal Radius (mm)	-10.68	-68.60 ~ –.65	0.65			

^a^***CCT*** central corneal thickness;

^b^***BMI*** body mass index;

^c^***WTW*** white-to-white corneal diameter

With *, *P* < 0.05

## Discussion

This study evaluated the distribution of ECD in Chinese school children. Previous studies have reported the ECD distribution in different populations, but most of the subjects were adults. For reference, we list the ECD distribution in subgroups closest in age from previous studies (Table 3) [14–27]. We can tell from the comparisons that the mean ECD in the present study was close to that among the Indian [14], Malay [20], Nigerian [23] young people from previous studies. Iranian young people seemed to have the lowest mean ECD among all these studies [17]. The mean ECD in the present study was lower than that among Pakistan [24] and Thai [27] teenagers, teenagers from a previous Chinese study [18] and Canadian [25] children. Whether the difference of euipments evaluating ECD or regions of participants made contributions keeps unknown, which necessitates further exploration. It is worth noting that most of these previous studies were not population-based and the sample size was relatively small. Large-scale studies are still needed to establish normal ECD values among different populations by age. Meanwhile, we must continue monitoring the ECD changes of each child in the NES cohort and it is necessary to depict the ECD-age curves of children and teenagers. The mean (± SD) WTW in this study was 12.14 ± 0.38 mm. Reports of normal WTW distribution were reviewed. The mean (± SD) WTW measured with Orbscan II in a German study [34] was reported as 11.71 ± 0.42 mm. An Iranian study [35] showed that the mean (± SD) WTW measured with Orbscan II in people aged 18–45 years was 11.65 ± 0.36 mm, while another Iranian study [36] found that the mean WTW measured with the LENSTAR/BioGraph in a 40- to 64-year-old population was 11.80 mm and that in the 40- to 44-year-old subgroup was 11.91 mm. A Chinese study [37] examined a large number of Chinese cataractous eyes and reported the mean WTW (± SD) measured with the ZEISS IOLMaster 700 as 12.00 ± 0.45 mm in males and 11.80 ± 0.50 mm in females aged 30- 40 years, close to our result. All of these studies suggested a decreasing trend of WTW with age. Thus, the relatively greater WTW in this study among Chinese school children is reasonable. The AL distribution in this study is similar to that in the population-based multicenter studies for Australian children [38] and European children [39], both measured with the Zeiss IOLmaster and that in the Anyang Childhood Eye Study for Chinese children [40] using Lenstar LS900.

**Table 3:** Studies of corneal endothelial cell density

Author	Year	Location	Age	Design	Sample Size	ECD (cells/mm^2^)
Rao SK[14]	2000	Chennai India	20–30 y		104 eyes	2782 ± 250
Müller A[15]	2002	Glasgow area and central Scotland UK	5–15 y		119 cases	3542 ± 510 range 2576–5316
Padilla MD[16]	2004	Makati Philippines	20–30 y		114 eyes/ 57 cases	2949 ± 270
Hashemian MN[17]	2006	Iran	20–30 y		102 eyes/ 102 cases	2407 ± 399
Yunliang S[18]	2007	Shantou China	11–20 y		100 eyes	3308 ± 356
Higa A[19]	2010	Kumejima Japan	40–49 y	population-based	827 eyes/ 827 cases	3031 ± 359
Mohammad-Salih PA[20]	2011	Kuantan Malaysia	20–30 y		49 eyes/ 49 cases	2783 ± 286
Galgauska S[21]	2013	Vilnius Lithuania	20–29 y		55 eyes/ 28 cases	2931 ± 371 range 2232–3610
Arici C[22]	2014	Istanbul Turkey	20–30 y		42 eyes/ 21 cases	2910.2 ± 365.9
Ewete T[23]	2016	Nigeria	20–30 y		81 cases	2860.70 ± 227.06
Islam QU[24]	2017	Karachi Pakistan	12–20 y		84 eyes/ 42 cases	3021.24 ± 312.24
Elbaz U[25]	2017	Toronto, Ontario, Canada	4-5y		24 eyes/ 24 cases	3746 ± 370 range 3145–5013
Abdellah MM[26]	2019	Egypt	20–30 y		89 eyes/ 89 cases	2933.75 ± 345.92 range 2843.5–2983.7
Tananuvat N[27]	2020	Chiang Mai Tailand	11–20 y		72 eyes/ 36 cases	2944.65 ± 231.95
This study	2021	Nanjing China	6.83–7.83 y	population-based	1171 eye/ 1171 cases	2875.34 ± 195.00 range 2066–3546

Age-related ECD decrease has been reported for several times. In this study, even within the narrow age range (6.83–7.83 years), ECD presented a decreasing trend towards age increase. However, age didn’t seem to be the only determinant of ECD. Within a relatively narrow age range, we explored the correlation between ECD and BW, general condition (age, gender and BMI) and ocular biometric parameters.

The results in this study presented positive correlation between ECD and BW and revealed that children born with low BW had significantly lower ECD than those born with normal BW and high BW. It is known that environments in utero such as maternal nutrition could affect fetal gene expression and developmental plasticity by epigenetic pathways [41]. BW may partly reflect antenatal development of the fetus and status of the newborn. To our knowledge, none of previous studies have reported the correlation between ECD and BW, though several studies demonstrated associations between BW and other corneal parameters. One study found an association between a lower BW and the following changes — steeper CR, smaller WTW, thinner CCT, and shorter AL [42]. Another study revealed that low BW is associated with thinner corneas in adolescence [43]. This study suggested that ECD had positive correlations with BW and CCT, which may explain the consistency to some extent. We speculate that prenatal growth development might affect corneal morphology in childhood, adolescence and adults.

This study showed that girls had higher mean ECD than boys. Another population-based Japanese study among adults also demonstrated that the mean ECD in women was significantly greater than that in men after adjusting for age [19]. Likewise, in the Scottish study, the ECD was slightly higher in the girls than that in the boys, although the difference was not statistically significant [15]. The Filipino study also showed that women had a mean ECD 7.8% higher than men [16]. Nevertheless, a previous Chinese study reported significantly higher mean ECD in men than that in women [19]. Other studies such as the Egyptian study [26] and the Iranian study [17] presented no significant differences in mean ECD between men and women.

BMI is a statistical index using a person's weight and height to provide an estimate of body fat. This study proved no significant correlation between ECD and BMI. However, a previous Chinese study found that ECD in children aged 3 years with dysplasia and obesity was lower than that in normal children. But this difference did not apply to other age groups (4–7 years, 7–10 years and 10–12 years) [44]. Another previous study reported that CCT had no correlation with BMI in adults [45]. Whether malnutrition or overnutrition during specific life stage exerts great impact on ECD needs further study.

ECD decreases at a relatively rapid rate during early childhood, which is considered mainly due to normal eye growth and increase in corneal size [7]. Whether corneal size influences ECD of school children was explored in this study. We even calculated SAC according to the area formula for spherical cap, but no correlation was found. A previous study accorded to our result in the matter of corneal diameter [46]. Other studies found ECD to be negatively correlated with corneal diameter in children [14, 15, 27]. However, one of these studies admitted that the decline in ECD after the age of 2 years is presumably caused by cell loss rather than corneal growth [15].

Controversial results towards the correlation between ECD and CCT have been reported by previous studies, including insignificant correlation [26, 27], negative correlation [47], and positive correlation [19, 21]. This study demonstrated the correlation to be positive, though CCT increase by 10 mm accompany ECD increase by 4 cells/mm^2^.

In the present study, BW, gender and CCT were found to be significantly associated with ECD. As CCT may be a parallel parameter with ECD, seeming also like the outcome, maybe we need to pay more attention to the innate quality, which means that we need to attach importance to the genetic factor and intrauterine environment. Besides, we also need to illuminate the rule for individual age-related ECD decrease and explore its influencing factors, which require longitudinal cohorts and mechanism research.

The strengths of this study include its population-based design, large sample size, and standardized examination protocols performed by an expert team. The age range of the subjects is relatively narrow, decreasing the impact of large age span on ECD distribution. Our analyses of associations with ECD are different from previous studies, by taking BW and anthropometric parameters into consideration. The exploration among normal children may help us understand the development of ECD and provide references for developing the prediction model for ECD decrease in the future. The limitation of this study is that BW collected through questionnaire may be biased. In addition, the correlation obtained from the cross-sectional study needs to be validated and deepened by further studies.

## Conclusions

In summary, our study provided normative data for the corneal endothelial cells and other ocular biometric parameters in Chinese school children aged 82 to 94 months. The ECD is positively correlated with BW and CCT, in which BW is a newly identified associated factor. Children born with low BW present significantly lower ECD than those born with normal BW and high BW. It is like that gender plays an important role in ECD distribution while girls have relatively greater ECD than boys.

## Data Availability

The datasets used during the current study are available from the corresponding author on reasonable request.

## Abbreviations

ECD: Corneal Endothelial Cell Density
BW: Birth Weight
BMI: Body Mass Index
CCT: Central Corneal Thickness
CR: Corneal Radius
AL: Axial Length
WTW: White-To-White Corneal Diameter
SAC: Surface Area of Cornea
SD: Standard Deviation
CI: Confidence Interval
MLR: Multiple Linear Regression
